# Infection-Associated Mortality During Induction Chemotherapy in Group B Intermediate-Risk Pediatric Burkitt’s Lymphoma

**DOI:** 10.7759/cureus.40365

**Published:** 2023-06-13

**Authors:** Syed Muhammad Ibne Ali Jaffari, Masooma Hashmi, Abdul Wasey Hashmi, Samaha Nisar, Hafsa Ashraf, Ghufran Tariq, Arslan Farooq, Javeria Awan, Syed Muhammad Jawad Zaidi, Mehwish Kaneez

**Affiliations:** 1 Pediatrics, Shalamar Medical and Dental College, Lahore, PAK; 2 Internal Medicine, Walsall Manor Hospital, Royal Wolverhampton NHS Trust, Walsall, GBR; 3 Pediatrics, Arif Memorial Teaching Hospital, Lahore, PAK; 4 Internal Medicine, Combined Military Hospital, Lahore, PAK; 5 Pediatrics, Rawalpindi Medical University, Rawalpindi, PAK; 6 Pediatrics, Holy Family Hospital, Rawalpindi, PAK; 7 Pediatric Oncology, Shaukat Khanum Memorial Cancer Hospital and Research Centre, Lahore, PAK

**Keywords:** bacterial infections, induction chemotherapy, infection-associated mortality, intermediate risk, burkitt's lymphoma

## Abstract

Background

Burkitt's lymphoma (BL) in the pediatric population has significant burden in developing countries. Infection-related complications during the induction chemotherapy phase pose a major challenge and contribute to high mortality rates due to a severely immunocompromised state. However, there is scarce data on the etiologies and optimal management strategies for infection-related mortality in pediatric BL patients, especially in developing countries like Pakistan.

Methods

This is a cross-sectional study that included a total of 116 pediatric patients with intermediate-risk BL. All patients were treated based on the Children’s Cancer and Leukaemia Group (CCLG) 2020 guidelines. Data on patient demographics, presenting symptoms, diagnosis, infectious etiologies, and outcomes were collected. Infection-related complications and mortality were monitored during the induction chemotherapy period. The results of relevant culture reports were tabulated and data were analyzed.

Results

Among the 116 included patients, 61.1% were males with a mean age of 4.83 ± 2.12 years. Abdominal BL was the most common anatomical location. During the induction period, 66 patients (56.9%) had culture-proven infections, resulting in 33 deaths (28.4%). Fever was the predominant presenting symptom in all patients, followed by vomiting (57.6%), loose stools (42.4%), and cough (18.2%). Neutropenic colitis, sepsis, pneumonia, and meningitis were among the diagnosed infections. Hospital-acquired bacterial infections, including multi-drug resistant gram-negative and gram-positive organisms, were the main cause of mortality, with fungal infections and cytomegalovirus viremia also identified in a few patients.

Conclusions

This study highlights the urgent need for improved management strategies in pediatric BL patients in Pakistan to reduce infection-related complications and mortality rates, emphasizing the importance of context-specific approaches for infection prevention and management.

## Introduction

Burkitt's lymphoma (BL) is a highly aggressive type of non-Hodgkin's lymphoma that commonly affects children [1]. It is prevalent in Africa and other regions of the world, including Pakistan [2]. In Pakistan, BL accounts for nearly 30% of all childhood cancers, with an approximate incidence rate of three cases per 100,000 children each year [3]. Despite the availability of standardized chemotherapy protocols for its treatment, the incidence of infection-related complications during induction chemotherapy remains high, particularly in developing countries where adequate nutrition, resources, and healthcare facilities are limited [4].

In the literature, infection-associated complications during induction chemotherapy in BL patients can lead to mortality rates as high as 10%-20% [3,4]. However, in developing nations like Pakistan, the incidence of infection-associated mortality during induction chemotherapy is reported approximately at 37% [5]. These statistics necessitate the need for optimal management strategies to reduce the incidence and severity of infections during induction chemotherapy.

Even though these complications significantly impact patient outcomes, there is a lack of data on the etiologies that contribute to infection-related mortality in pediatric BL patients in developing countries [6]. Therefore, our study aims to address this gap in knowledge by investigating the incidence, etiological factors, and management strategies for infection-related mortality in pediatric BL patients. Our study also aims to give appropriate recommendations to mitigate these crises. The identification of the causes of infection-related complications and recommendations on optimized management strategies will aid to reduce mortality and improve overall survival.

## Materials and methods

This cross-sectional study was conducted at the Department of Pediatrics, Holy Family Hospital, Rawalpindi, Pakistan between August 2020 till March 2023. All pediatric patients categorized with group B intermediate risk BL were included in the study. Children with less than one year of age, low-risk disease, and stage four disease with central nervous system (CNS) involvement were excluded from the study. Computed records and patient files were utilized for the collection of data regarding patient demographics, culture reports, infectious etiologies, diagnoses, and outcomes. The study was ethically approved by the Institutional Review Board at Rawalpindi Medical University (approval number: 2021-PM-15340).

The risk stratification and staging of all patients were completed according to the Children's Cancer and Leukaemia Group (CCLG) 2020 guidelines. Baseline bone marrow biopsy and diagnostic lumbar puncture were used to determine bone marrow and CNS involvement respectively. According to CCLG guidelines, patients with incompletely resected stage one or two diseases, residual nodal disease, and stage three non-bulky disease without CNS or bone marrow involvement were categorized as group B intermediate risk.

All newly diagnosed BL patients were admitted for initial stabilization and initiation of treatment. Tumor lysis profile monitoring was performed for the first 24-48 hours, followed by baseline scans, and the start of pre-phase chemotherapy for six days which included vincristine, cyclophosphamide, and prednisolone (COP protocol). Thereafter, evaluation of tumor response is observed on day 7 with a reassessment computed tomography scan. Thereafter, the first cycle of induction chemotherapy was started. Chemotherapy agents used during two cycles of induction were methotrexate, doxorubicin, vincristine, intrathecal methotrexate, and prednisolone (COPADM). The COPADM protocol lasted for six days with two intrathecal chemotherapy on day two and day six.

After the end of the first and second cycles of induction chemotherapy, patients were counseled to report to pediatric emergency in case of fever. A sudden increase in temperature to 38.3°C was characterized as fever. All patients with fever had a comprehensive physical examination, relevant laboratory tests, and imaging for appropriate localization of the cause. Additionally, they were given a dose of empirical piperacillin and tazobactam within one hour of the presentation due to suspicion of febrile neutropenia. On the initial complete blood count, if the absolute neutrophil count was less than 1,000, then patients were admitted and were managed on the line of febrile neutropenia. Confirmation of infection was made using final reports of relevant cultures (blood, urine, sputum, tracheal aspirates, or tissue specimens). Central and peripheral blood cultures were drawn on admission and were repeated if required. Ideally, the induction period (including one pre-phase chemotherapy cycle and two induction chemotherapy cycles) consisted of approximately 35 days but was prolonged in many patients due to prolonged febrile neutropenia. All deaths that occurred during the induction period were included in the analysis. The data were analyzed using the Statistical Package for Social Sciences (SPSS) version 25 (IBM Corp., Armonk, NY). Categorical variables were presented as frequencies and percentages, while numerical variables were presented as means and standard deviations.

## Results

A total of 116 patients were included in the study of which 74 (61.1%) were males. The mean age of the study participants was 4.83 ± 2.12 years. Most of the patients had abdominal BL at presentation. The demographic details of the study participants are shown in Table 1.

**Table 1:** A tabulation of demographical details of patients.

Parameters		Frequency (n = 116)	Percentages
Gender	Male	74	63.8%
	Female	42	36.2%
Age	1-5 years	81	69.8%
	More than 5 years	35	30.2%
Anatomic location of the tumor	Abdomen	108	93.1%
	Facial/jaw	8	6.9%
Infections	Yes	66	56.9%
	No	50	43.1%
Outcome	Dead	33	28.4%
	Alive	83	71.6%

During the defined induction period, a total of 66 patients had culture-proven infection of which 33 (28.4%) patients died. All admitted patients had severe neutropenia on initial presentation. Fever was the most common presenting complaint in the emergency, followed by loose stools, cough, flu-like symptoms, increased work of breathing, and vomiting. Further details regarding presenting symptoms and diagnosis are shown in Table 2.

**Table 2:** Details regarding presenting symptoms and diagnosis.

Parameters		Frequency (n = 33)	Percentages
Presenting symptoms	Fever	33	100%
	Loose stools	14	42.2%
	Cough	6	18.2%
	Flu	3	9.1%
	Vomiting	19	57.6%
	Tachypnea	5	15.1%
	Seizures	2	6%
Diagnosis	Neutropenic Colitis	14	42.2%
	Sepsis	11	33.3%
	Pneumonia	6	18.2%
	Meningitis	2	6%
Tumor lysis profile at the initiation of treatment	Normal	21	63.6%
	Deranged	12	36.4%

All the mortalities were associated with infections. Out of 33 patients who died, 26 blood cultures were positive for bacterial microorganisms. Of these 26 blood cultures, 19 had gram-negative bacterial infections, and seven acquired gram-positive bacterial infections. Five patients tested positive for fungal infections while two patients had cytomegalovirus viremia. Further details regarding infectious etiologies are delineated in Table 3.

**Table 3:** An elucidation of isolated infectious organisms in our patients.

Organism Isolated	Mode of diagnosis	Frequency (n = 33)	Percentages
Multidrug resistant Escherichia coli	Blood cultures	6	18.2%
Multidrug resistant Klebsiella	Blood cultures	4	12.1%
Pseudomonas	Tracheal aspirates	1	3%
Multidrug resistant Acinetobacter	Blood cultures	1	3%
Stenotrophomonas	Tracheal aspirates	2	6%
Streptococcus pneumoniae	Cerebrospinal fluid culture	2	6%
Burkholderia species	Urine culture	2	6%
Multidrug resistant Escherichia coli	Urine culture	3	9.1%
Invasive Aspergillosis	Bronchoscopy/computed tomography scan	2	6%
Fluconazole resistant Candidemia	Blood culture	3	9.1%
Methicillin-resistant Staphylococcus aureus	Blood cultures	3	9.1%
Vancomycin-resistant Staphylococcus aureus	Wound swab	1	3%
Vancomycin-resistant enterococcus	Stool cultures	1	3%
Cytomegalovirus	Polymerase chain reaction	2	6%

## Discussion

Even with comprehensive guidelines on supportive care in pediatric oncology patients, favorable outcomes are difficult to achieve in pediatric BL patients [7]. Lack of resources, malnutrition, poor hygienic measures, nosocomial infections, lack of adherence to antimicrobial stewardship programs, and irrational use of antimicrobials are a few reasons reported in the literature for an increase in infection-associated mortality among such patients [6,8] and our study results are consistent with these findings.

Reports from various developing countries have indicated a treatment-related infectious mortality rate ranging from 20% to 30% [9,10]. In a separate study conducted in India, six (20.6%) out of 29 BL patients in treatment group B, succumbed to neutropenic sepsis following induction chemotherapy [11] Our findings align with these reported statistics, highlighting the heightened mortality rate in our region. Conversely, a study conducted in Central America revealed a relatively lower end-of-induction mortality rate of 8.8% [12]. Similarly, reports from national cancer registries in developed countries consistently document lower mortality rates [8]. The lower mortality rates reported in developed countries emphasize the importance of comprehensive infection prevention and management approaches [4]. Overall, these findings emphasize the critical role of infections in determining the prognosis and survival outcomes in pediatric BL patients. This necessitates further research and targeted interventions to mitigate these risks.

Literature shows that aggressive chemotherapy regimens, frequent hospitalizations, and intense medical interventions in pediatric BL patients predispose them to various hospital-acquired infections [5,13]. Multidrug-resistant gram-negative organisms including Klebsiella, Pseudomonas, and Escherichia coli are major culprits for severe gram-negative neutropenic sepsis and death in our patients. Similar reports are obtained from other studies as well [11,14,15]. Central venous catheters, urinary catheters, respiratory equipment, poor hand hygiene, and contaminated surfaces remain the major culprits for the spread of these organisms [15]. Preventing and managing hospital-acquired infections require a multidisciplinary approach that includes strict adherence to infection control practices, such as hand hygiene, proper sterilization of medical equipment, and appropriate use of antimicrobial agents. Additionally, regular surveillance for infections, prompt diagnosis, and targeted treatment based on culture results are essential to prevent complications, reduce antibiotic resistance, and improve patient outcomes.

BL represents a significant public health challenge especially in developing countries like Pakistan, and infectious complications during chemotherapy are a major concern [9]. Improving supportive care measures and the overall quality of care in pediatric oncology centers is essential to improve overall outcomes for these patients [16]. Moreover, the development of new treatment approaches that are less toxic chemotherapy protocols with equal efficacy having fewer treatment-related complications is an important consideration for malnourished children in developing countries [8].

The collection of data from a single center and having a cross-sectional study design are a few limitations of our study. Nationwide data collection from multiple centers would have improved the results of our study. Nonetheless, our study highlights important points regarding the management of pediatric BL. Our study also emphasizes the need for local guidelines for the management of infections in neutropenic sepsis in such patients. Further research is needed to better understand the epidemiology, pathogenesis, and treatment outcomes of BL in this population, as well as to identify effective strategies to reduce the risk of infections and improve overall survival. Through the results of our study, we hope to contribute to the growing body of knowledge on the management of infections in pediatric BL and ultimately improve the prognosis and quality of life for such patients in developing countries like Pakistan.

## Conclusions

BL is a major public health concern in Pakistan and other parts of the world. The high incidence of infection-related complications and mortality in BL patients in Pakistan highlights the urgent need for better management strategies to improve patient outcomes. The findings of our study emphasize the urgent need for targeted and optimized management strategies to effectively reduce infection-related complications and mortality rates during the induction chemotherapy phase. Further research is essential to address the unique challenges faced by pediatric BL patients in resource-limited settings, with a particular focus on developing context-specific approaches for infection prevention and management, aiming to significantly improve patient outcomes and overall survival.
